# Editorial: Transforming special care dentistry: frameworks for education, clinical excellence and technological innovation

**DOI:** 10.3389/froh.2026.1974373

**Published:** 2026-09-09

**Authors:** Gustavo Fabián Molina, Mariana Morales-Chávez, Susanne Krämer, Koichiro Matsuo, Jacobo Limeres Posse, Jorma I. Virtanen, Mathew Lim

**Affiliations:** 1 Special Care Dentistry, Faculty of Health Sciences, Universidad Católica de Córdoba, Córdoba, Argentina; 2 Dental Materials Department, The Dental Faculty, Universidad Nacional de Córdoba, Córdoba, Argentina; 3 Dental Research Center, Universidad Santa María, Caracas, Venezuela; 4 Special Care Dentistry Unit, Faculty of Dentistry, University of Chile, Santiago, Chile; 5 Department of Oral Health Sciences for Community Welfare, Graduate School of Medical and Dental Sciences, Institute of Science Tokyo, Tokyo, Japan; 6 Special Care Dentistry, School of Medicine and Dentistry, Universidad de Santiago de Compostela, Santiago de Compostela, Spain; 7 Faculty of Medicine, University of Bergen, Bergen, Norway; 8 Saveetha Dental College and Hospitals, Saveetha University, Chennai, Tamil Nadu, India; 9 Melbourne Dental School, University of Melbourne, Melbourne, VIC, Australia; 10 School of Translational Medicine, Monash University, Clayton, VIC, Australia

**Keywords:** dental education, disabilities, oral health inequalities, oral health promotion, special care dentistry

This Research Topic brings together a diverse yet highly complementary body of work that reflects the continuing maturation of Special Care Dentistry (SCD) as a distinct discipline. SCD encompasses the delivery of oral healthcare for individuals whose physical, intellectual, medical, psychological or social conditions require modifications to conventional dental care in order to achieve equitable oral health outcomes (1). Collectively, the nine articles move beyond the traditional perception of SCD as a discipline focused primarily on overcoming barriers to care, instead demonstrating how structured education, evidence-based clinical practice, service innovation, and interdisciplinary collaboration can support equitable access to high-quality oral healthcare for people with disabilities, complex medical conditions, and other vulnerable populations. Viewed together, these contributions illustrate the evolution of SCD from a discipline primarily focused on providing access to care towards one increasingly defined by competency-based education, integrated clinical pathways, patient-centred care and evidence-informed service delivery.

Although the scientific literature in SCD has expanded considerably over the past two decades, important gaps remain, particularly regarding international collaboration, multicenter research and the generation of high-quality evidence across diverse healthcare settings. A particular strength of this collection lies in its international scope: the contributions originate from diverse regions of the world, including the Asia-Pacific region, Europe, and Latin America, reflecting a broad range of educational systems, healthcare structures, and clinical realities. This diversity provides a unique opportunity to examine SCD through multiple lenses while identifying common challenges and shared aspirations. Despite differences in resources, policies, and service delivery models, the studies collectively highlight a global commitment to improving oral health outcomes for people with disabilities, complex medical conditions, rare diseases, and other vulnerable populations. The international perspectives presented here enrich the evidence base and demonstrate that innovation in SCD is not confined to a single region but is emerging across diverse cultural and healthcare contexts, particularly in countries that are new to recognising the specialty or who are in the process of developing it (2). It shows a real recognition of the need for oral health equity and the spreading of this message to all corners of the globe.

Many countries have included teaching and learning in SCD within their curricula, however, great variation between continents and countries exist. This Research Topic highlights the importance of educational frameworks in preparing the future workforce. The studies by Lim et al. and Koh et al. address this challenge from complementary perspectives. The survey of 31 dental schools across the Asia-Pacific region provides an important overview of undergraduate SCD education and emphasizes both progress and variability in the implementation of the International Association for Disability and Oral Health (iADH) curriculum guidance (3, 4). While most institutions reported achieving many learning outcomes, challenges such as limited clinical exposure and shortages of trained faculty remain significant barriers. Expanding on these findings, Koh et al. present a comprehensive, evidence-informed curriculum framework for oral health therapy programs, proposing competency domains and educational pathways that support the progressive development of knowledge, skills, and confidence in providing disability-inclusive care. Across these diverse settings, four recurring themes emerge: workforce development, evidence-informed clinical practice, patient-centred models of care and service innovation. These themes provide the framework for understanding the contributions presented in this Research Topic.

Ensuring that graduates achieve meaningful clinical competence requires assessment methods that are as robust as the curricula themselves. Educational innovation is further explored by Morlock et al., who examined the use of structured oral examinations (SOEs) as a tool for competency assessment in SCD. Their findings suggest that standardized assessment approaches can consistently distinguish between different levels of clinical competence, even when expert assessors are limited. As educational programs seek to expand SCD training globally, reliable and scalable methods for evaluating clinical reasoning and decision-making will become increasingly important. Furthermore, effective training in SCD depends not only on the content of the curriculum, but also on meaningful clinical experience, competent trainers and structured assessment.

Beyond education, several studies in this collection provide valuable insight into the complexity of patients requiring special care services. The retrospective hospital audit by Lee et al. documents the substantial oral health burden and case complexity encountered in a tertiary referral center. A large proportion of patients were classified as severe or extreme complexity, with many requiring advanced pharmacological behavioral support. These findings point to the need for integrated care pathways and more effective transitions between primary, secondary, and tertiary services to prevent oral disease progression and reduce avoidable treatment complexity.

The pilot mixed-methods study of Austrian patients with epidermolysis bullosa (EB) offers an important perspective on the oral health challenges associated with rare diseases. By combining clinical assessments with patient-reported outcomes, the authors demonstrate the profound impact of EB—particularly recessive dystrophic EB—on oral health, oral function, and quality of life (5). Importantly, the study carried out by Veliz et al. illustrates the broader evolution of SCD towards person-centred models of care in which clinical decision-making incorporates not only disease characteristics but also patient experience, function and quality of life.

Adapting the clinical environment to patients' individual sensory and communication needs has become an increasingly important component of contemporary SCD. The evaluation of the Educational Sensory Based Approach (ESBA) for children with autism spectrum disorder demonstrates how tailored behavioral interventions can significantly improve cooperation during dental treatment. The findings presented by Corridore et al. support the growing recognition that environmental adaptations, sensory considerations, and individualized communication strategies can reduce reliance on general anesthesia while improving the patient's experience. Such approaches exemplify the shift toward proactive and personalized models of care within SCD.

Despite the growing emphasis on behavioral and environmental approaches, pharmacological management remains indispensable for many individuals with complex healthcare needs (6). Two studies in this collection contribute important evidence in this area. Sato et al. describe the medication profiles of patients receiving dental treatment under sedation or general anesthesia, revealing high rates of medication use and polypharmacy, particularly involving antiepileptic and antipsychotic drugs. Their findings underscore the need for clinicians to understand potential pharmacological interactions and associated risks when planning dental treatment. Together, these studies illustrate that safe pharmacological management depends both on understanding patient complexity and on delivering sedation within appropriately organized clinical systems.

Rather than representing isolated advances, these studies collectively illustrate the emergence of a more integrated model of SCD in which education, clinical care, service organisation and patient-centred practice are increasingly interconnected. They span the educational continuum from curriculum design to competency assessment; they explore the epidemiology and complexity of populations requiring specialized care; they evaluate behavioral, preventive, and pharmacological approaches to treatment delivery; and they highlight the importance of integrating patient-centered outcomes into clinical decision-making.

Most importantly, these studies collectively advance the central objective of this Research Topic: the systematization of SCD. By developing structured educational frameworks, standardizing assessment methods, generating evidence to inform clinical pathways, and evaluating innovative models of care, the authors contribute to the ongoing transformation of SCD into a robust, evidence-driven discipline. While important challenges remain,—including workforce development, service integration, and equitable access to care—the work presented here demonstrates that meaningful progress is being achieved across multiple domains.

The proportion of people with significant disabilities is high: 16% of the global population experiences significant disability according to the WHO (7). As populations age, the prevalence of disability increases, and healthcare systems confront growing complexity, the need for well-trained professionals, integrated services, and innovative care models will continue to expand. Equally important, the global representation of authors and clinical settings within this collection demonstrates that the advancement of SCD is a truly international endeavor. The diversity of experiences presented—from tertiary referral centers and university programs to specialized outpatient clinics and rare disease services—illustrates how shared challenges can inspire locally adapted solutions with broader relevance. By learning from one another across geographical and professional boundaries, the SCD community can continue building a more equitable, evidence-based, and globally connected future for oral healthcare.

In all, these contributions suggest that SCD is no longer defined simply by the populations it serves but increasingly by the principles that underpin its practice. Competency-based education, person-centred care, multidisciplinary collaboration, evidence-informed decision-making and integrated service delivery are becoming core elements of high-quality dentistry more broadly. In this sense, the advances presented in this Research Topic extend beyond SCD itself and offer valuable lessons for the future development of oral healthcare systems. We hope that the evidence, experiences, and perspectives presented in this collection will stimulate further research, encourage international collaboration, and support policymakers, educators, clinicians, and researchers in advancing a future where high-quality oral healthcare is accessible to all, regardless of ability or circumstance.
